# Cellulose Biomaterials for Tissue Engineering

**DOI:** 10.3389/fbioe.2019.00045

**Published:** 2019-03-22

**Authors:** Ryan J. Hickey, Andrew E. Pelling

**Affiliations:** ^1^Department of Physics, STEM Complex, University of Ottawa, Ottawa, ON, Canada; ^2^Department of Biology, University of Ottawa, Ottawa, ON, Canada; ^3^Institute for Science Society and Policy, University of Ottawa, Ottawa, ON, Canada; ^4^SymbioticA, School of Human Sciences, University of Western Australia, Perth, WA, Australia

**Keywords:** cellulose, nanostructure, biomaterials, biocompatibility, mechanics

## Abstract

In this review, we highlight the importance of nanostructure of cellulose-based biomaterials to allow cellular adhesion, the contribution of nanostructure to macroscale mechanical properties, and several key applications of these materials for fundamental scientific research and biomedical engineering. Different features on the nanoscale can have macroscale impacts on tissue function. Cellulose is a diverse material with tunable properties and is a promising platform for biomaterial development and tissue engineering. Cellulose-based biomaterials offer some important advantages over conventional synthetic materials. Here we provide an up-to-date summary of the status of the field of cellulose-based biomaterials in the context of bottom-up approaches for tissue engineering. We anticipate that cellulose-based material research will continue to expand because of the diversity and versatility of biochemical and biophysical characteristics highlighted in this review.

## Introduction

A fundamental understanding of the nanoscale details of the environment is essential for designing biomaterials that mimic the natural cellular milieu. Many features of the local environment have profound influences on cell adhesion, proliferation, maturation, and differentiation. As such, small differences in nanostructure can have macroscale impacts on tissue function. In this review, we highlight the importance of nanostructure of cellulose-based biomaterials to allow cellular adhesion, the contribution of nanostructure to macroscale mechanical properties, and several key applications of these materials for fundamental scientific research and biomedical engineering. Cellulose is a diverse material with tunable properties and can be applied to systems with vastly different biochemical and biophysical environments. It should be noted that many polymers can be functionalized; therefore, polymers in general are diverse materials. Cellulose-based biomaterials offer some important advantages over conventional synthetic materials and show great promise to advance the frontier of scientific knowledge. Here we provide an up-to-date summary of the status of the field of cellulose-based biomaterials in the context of bottom-up approaches for tissue engineering. We anticipate that cellulose-based material research will continue to expand because of the diversity and versatility of biochemical and biophysical characteristics highlighted in this review.

## Cellular Attachment at the Nanoscale

### Cell Adhesion

**Figure 1:**
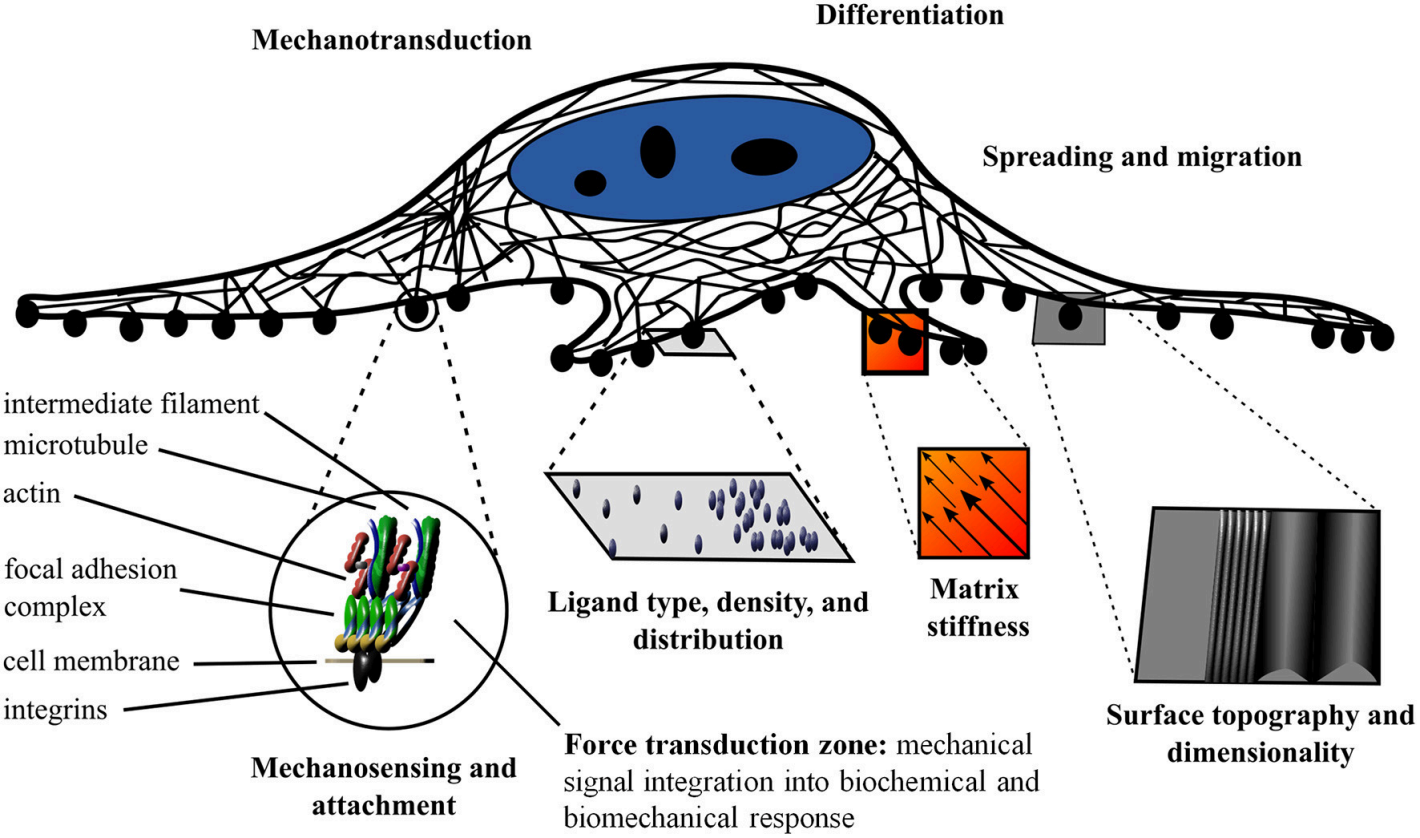
Schematic of the influence of the local physical and biochemical environment on cell fate and function. Mechanosensing and mechanotransduction are mediated by cell adherence to the substrate via integrins and the interaction of focal adhesions and the cytoskeleton. The ligand type, density, and distribution as well as the matrix stiffness, surface topography, and dimensionality provide distinct cues to the cell and elicit specific responses.

It is well-established that the extracellular matrix (ECM) not only allows for cell attachment, but also provides biochemical and biophysical cues to the nascent cells and tissues (Jaalouk and Lammerding, 2009; Holle and Engler, 2011; Plotnikov and Waterman, 2013; Gautrot et al., 2014; Gregor et al., 2014; Wickström and Niessen, 2018). In order for cells to sense and respond to their physical environment, they must first establish a physical connection (Lazarides and Burridge, 1975; Geiger et al., 1980). This physical connection is often mediated by the integrin protein complexes that recognize the widely conserved tripeptide recognition sequence of the ECM (Ruoslahti and Pierschbacher, 1987). It is important to note that the integrin based attachment to the RGD motif is not the only method of attachment; however, it has been studied in depth. The integrin receptor complexes constitute a variable class of proteins that are heterodimeric with two membrane-spanning subunits (Hynes and Destree, 1976; Horwitz et al., 1986; Ruoslahti and Pierschbacher, 1987; Chen et al., 2003). The integrin receptors are linked to the cytoskeleton by focal adhesion complexes (Heath et al., 1978). The focal adhesions are multi-protein complexes organized in specific strata. The base layer establishes a membrane-apposed integrin signaling layer (Kanchanawong et al., 2010). The basal layer is followed by the force transduction zone (cytoskeletal adaptors), and the upper most layer mediates the cytoskeleton regulatory protein connections (Kanchanawong et al., 2010). Evidently, the physical cues of the environment on the nanoscale elicit specific responses and dictate cellular function (Engler et al., 2004; Al-Rekabi and Pelling, 2013; Higuchi et al., 2013; Murray et al., 2014; Knight and Przyborski, 2015; Ravi et al., 2015; Hickey and Pelling, 2017) A schematic of the cell attachment is presented in Figure 1. Specifically, the topography, adhesion chemistry and localization, and mechanics play crucial roles in regulating cell fate and function (Harris et al., 1980; Dalby et al., 2002, 2007). The cell adhesion machinery along with the hydrophilic hydroxyl moieties of the cellulose and specialized cellulose binding domains allow cells to attach to cellulose (Tormo et al., 1996; Levy and Shoseyov, 2002; Rakotoarivonina et al., 2002).

### Effects of the Nanoenvironment

The biochemistry of the surrounding environment has effects on cell morphology, adhesion, and proliferation (Liu et al., 2014). Cell attachment is dependent on the type of ligand in the ECM and the spacing of the ligand. Cells can modulate their environment by secreting ECM proteins (Muth et al., 2013). The nanoscale distribution of adsorbed proteins in both area and clustering affects cell adhesion (Hiraguchi et al., 2014). The ligand density at the nanoscale level and integrin clustering affects spreading, focal contact formation, stress fiber arrangement, cell motility, and filopodia and lammelipodia development (Maheshwari et al., 2000; Amschler et al., 2014). For example, different ligand densities give rise to the apparent paradoxal enhanced tumor growth with RGD analogs (Amschler et al., 2014). The paradoxal enhanced tumor growth with RGD analogs is the phenomenon where tumors grow and spread where contact and adhesion are suppressed. It occurs as the density is shifted from the optimal density to the permissive density region (Amschler et al., 2014). The permissive density mimics receptor blocking drugs and sheds light on the paradoxal enhanced tumor growth with RGD analogs (Amschler et al., 2014). Nanostructure dimensions are important in addition to substrate rigidity (Kuo et al., 2014). There has been debate over whether matrix stiffness or ligand density regulates differentiation; however, after decoupling the surface chemistry and stiffness effects, it was elucidated that matrix stiffness is an independent regulator of stem cell differentiation (Harris et al., 2013; Ye et al., 2015). Both the matrix stiffness and nanoscale spatial organization of the ligands direct stem cell fate (Engler et al., 2004, 2006; Harris et al., 2013; Muth et al., 2013; Amschler et al., 2014; Ye et al., 2015). The physical cues are not restricted to elasticity; local changes in surface structure, hydrophobicity, roughness, and charge density lead to different cell adhesion and proliferation properties (Kiroshka et al., 2014; Alshehri et al., 2016; Pedraz et al., 2016). Taken together, there is an integrated response to external and internal stimuli on the nanoscale, both physical and biochemical in nature (Wickström and Niessen, 2018) (Figure 1). Mimicking the complexity of the nanoscale environment is essential for tissue engineering.

## The Potential of Cellulose as a Biomaterial

### Suitability for Biomaterials and Scaffolding

In order to replicate important aspects of the *in vivo* environment, biomaterials must be biocompatible and contain specific mechanical, biochemical, and physical properties. As a polymer of glucose subunits (Haworth, 1928), cellulose is an ideal candidate for biomaterial manufacturing because of its tunable chemical, physical, and mechanical properties (Domingues et al., 2014; Mohite and Patil, 2014; Courtenay et al., 2018a,b). The source material is abundant in nature and is easily produced; consequently, cellulose-based materials constitute a low cost platform for tissue engineering. Biocompatibility, bioactivity, and biomechanics are three integral requirements of any biomaterial; cellulose-based biomaterials satisfy each of these criteria (Domingues et al., 2014; Mohite and Patil, 2014; Courtenay et al., 2018b). The reader is encouraged to consider inertness as a requirement as well. A biologically inert material is desired to eliminate foreign body responses. Nevertheless, completely biologically inert materials do not exist. Hence we argue proper bioactivity is a key requirement to elicit certain responses. To that end, biodegradability is another feature to be considered. Cellulose is not biodegradable in humans. Thus, the regenerated new tissue cannot take the place of the cellulose. There is significant debate on the use of degradable materials compared to permanent constructs. Both have advantages and drawbacks. In the case of cellulose, a possible drawback is that the cellulose will occupy space that the tissue cannot. A potential advantage of using this long-lasting material is continuous structural support. Cellulose-based materials can be derived from bacteria, tunicates, and plants (Domingues et al., 2014; Mohite and Patil, 2014; Courtenay et al., 2018b). The scaffolds can be naturally derived or synthetically manufactured. The nanoscale presentation of functional chemical groups and the associated physical properties are dependent on the source material along with the fabrication process.

### Molecular and Crystal Structure

**Figure 2:**
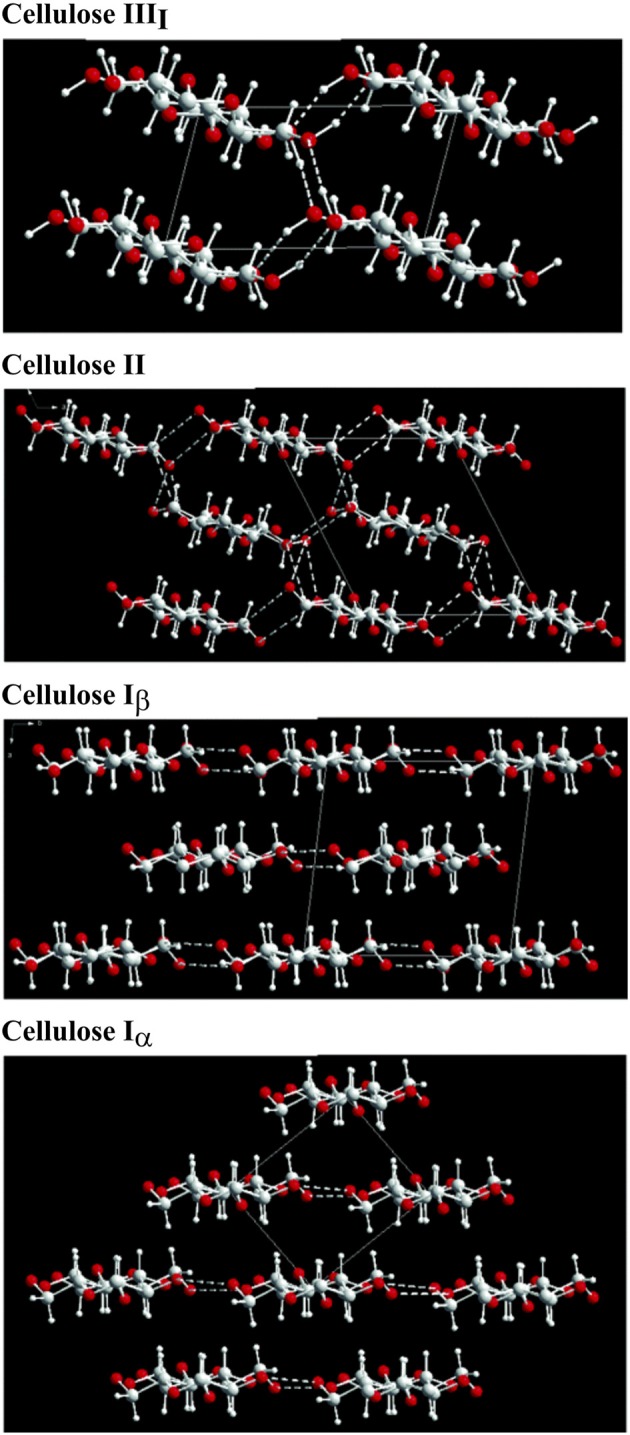
Crystal structure cellulose strands and the corresponding major hydrogen bonding arrangements (Wada et al., 2004). Copyright 2004. Reproduced with permission from Elsevier Inc.

The structure of cellulose is hierarchical, and the associated physical properties are a consequence of the different structural allomorphs and assemblies of elementary microfibrils; (Kroon-Batenburg and Kroon, 1997; Gibson, 2012; Hayakawa et al., 2017). The allomorphs of cellulose arise from the different arrangements of the chains of β-(1,4′)-D-glucopyranose monmers (Nishiyama et al., 2002, 2003b; Pérez and Mazeau, 2004; Wada et al., 2004; Miyamoto et al., 2009; Yamane et al., 2013). Cellulose I, the native form of cellulose, is defined by specific intrachain, interchain, and intersheet hydrogen bonding and van der Waals interactions). In cellulose I, the chains run parallel to one another and are present in two main crystal structures: cellulose I_α_ (triclinic) and cellulose I_β_ (monoclinic) (Sarko and Muggli, 1974; Moon et al., 2011). Although both crystalline forms of cellulose I are present together, cellulose I_β_ is the pre-dominant form in higher plants, whereas cellulose I_α_ is in abundance in bacterial and algal cellulose (Atalla and VanderHart, 1984). In contrast to the naturally occurring cellulose I, cellulose II is a synthetic material. Although the fundamental cellobiose subunits are the same as in cellulose I, the interchain and intersheet hydrogen bonding are altered due to its antiparallel arrangement (Sarko and Muggli, 1974; Kroon-Batenburg and Kroon, 1997; Nishiyama et al., 2002, 2003b; Pérez and Mazeau, 2004; Kim et al., 2006; Miyamoto et al., 2009; Gross and Chu, 2010; Yamane et al., 2013; Hayakawa et al., 2017). Cellulose II is derived from cellulose I with alkali treatment and is an irreversible transition (Oudiani et al., 2011a,b; Gupta et al., 2013; Jin et al., 2016). The third class of cellulose structure, cellulose III, is characterized by hydrogen bonding between separate sheets (Wada, 2001; Pérez and Mazeau, 2004; Wada et al., 2004, 2009). Moreover, cellulose III can be arranged in the parallel direction (cellulose III_I_) or the antiparallel direction [cellulose III_II_)]. Cellulose III can be made by exposing cellulose I (parallel) or cellulose II (antiparallel) to liquid ammonia and amine treatment). The formation of cellulose III is a reversible reaction; restoration to cellulose I and II, respectively can be achieved with thermal treatment (Wada, 2001; Pérez and Mazeau, 2004). The different crystal structures and hydrogen bonding arrangements are highlighted in Figure 2.

### Nanostructure Dictates Physical Properties

The crystal structure and degree of crystallization has a profound effect on the mechanical and physical properties (Nishino et al., 1995; Kroon-Batenburg and Kroon, 1997; Gibson, 2012). Importantly, the different hydrogen bonding results in different Young's moduli: cellulose I = 138 GPa, cellulose II = 88 GPa, cellulose III_I_ = 87 GPa, and cellulose III_II_ = 58 GPa (Nishino et al., 1995). In addition to the different moduli, the stability of the different allomorphs is also variable; in general, the order of decreasing stability is cellulose I, II, III, then amorphous (Igarashi et al., 2007, 2011; Kim et al., 2010; Nishiyama et al., 2010; Wada et al., 2010; Chundawat et al., 2011; Mittal et al., 2011). In nature, cellulose exists as a mixture of crystalline and amorphous structures, plausibly organized in a fringed fibril arrangement (Hearle, 1957). The combination of crystalline and amorphous elements results in the observed leveling off degree of polymerization, wherein the amorphous regions depolymerize before the crystalline domains (Battista et al., 1956; Hearle, 1957; Nishiyama et al., 2003a). The stability and degradation rates are crucial factors for the design of biomaterials (Modulevsky et al., 2016). These amorphous regions reduce the stiffness of the microfibril. Elementary microfibrils aggregate to form larger bundles; hence, an even greater diversity of mechanical and physical properties is available because of the different microfibril arrangements of the source materials. As such, the nanoscale properties such as the disorder and coalescence ratio along with the surface chemistry dictate the macroscopic properties. Cellulose-based materials have been selected for use as biomaterials because of their diverse and tunable properties (Domingues et al., 2014; Mohite and Patil, 2014; Courtenay et al., 2018b).

## Bacterial and Plant Cellulose

### Natural vs. Synthetic Materials

**Figure 3:**
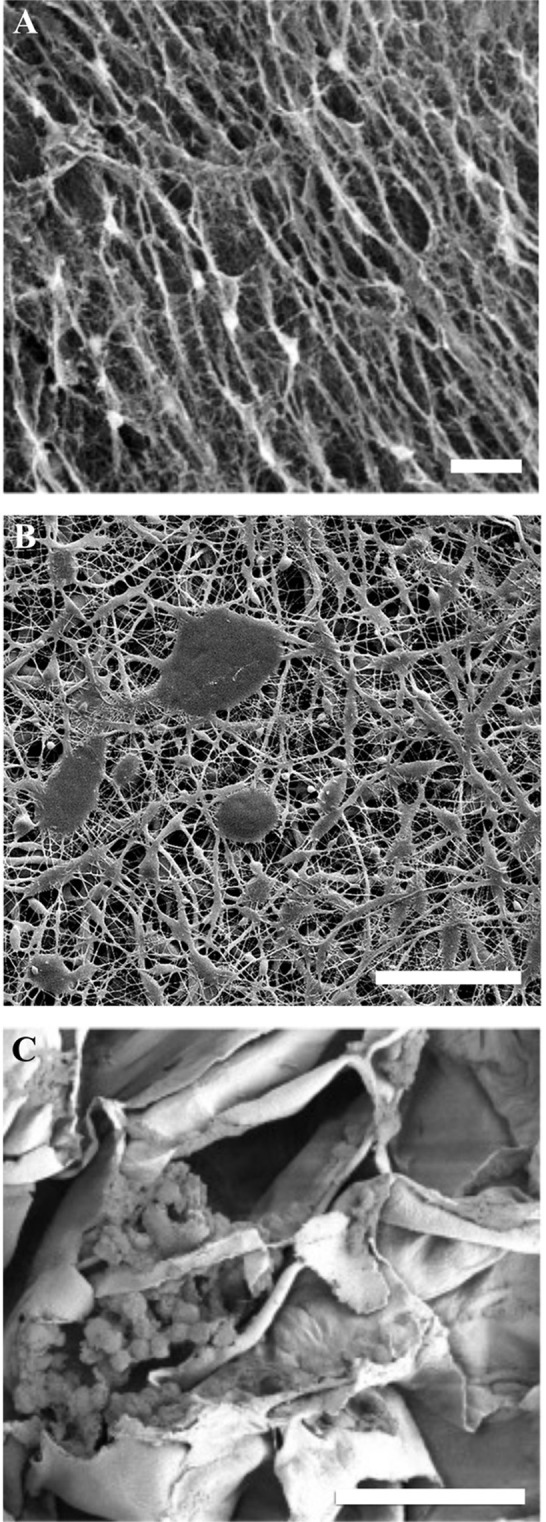
SEM images of cellulose biomaterials. **(A)** NIH 3T3 cells cultured on a bacterial cellulose film, scale = 10 μm (Fu et al., 2013). Copyright 2013. Reproduced with permission from Elsevier Inc. **(B)** Schwann cells cultured on a synthetic electrospun cellulose matrix, scale = 100 μm (Naseri-Nosar et al., 2017). Copyright 2017. Reproduced with permission from Elsevier Inc. **(C)** C2C12 cells cultured on decellularized apple cellulose scaffolds, scale = 50 μm. (Modulevsky et al., 2014) Copyright 2014. Reproduced with permission from PLOS.

In general, cellulose-based materials can be divided into naturally derived and synthetic materials (Figure 3). As shown in Figure 3, naturally derived (such as bacterial and plant based scaffolds) as well as synthetic materials can be used as biomaterials. The naturally derived celluloses have a cellulose I crystal structure (Pérez and Mazeau, 2004; Kim et al., 2010), whereas the synthetic materials are cellulose II and III (Wada et al., 2004; Jin et al., 2016). Pulp and paper is an entire industry dedicated to refining the production process and modifications of synthetic cellulose (Torres et al., 2012). A discussion of the vast processing of synthetic cellulose is beyond the scope of this review; however, we highlight that the crystal structure is different, and the different crystal structures lead to significantly different physical properties.

### Nanostructure Differences

Although bacterial and plant based cellulose are both type I, the slight differences in the crystal structure and microfibril arrangements lead to considerably different material properties (Nishino et al., 1995; Pérez and Mazeau, 2004; Kim et al., 2010; Nishiyama et al., 2010). The significant differences between bacterial and plant based cellulose are the purity, water retention, mechanical characteristics, crystallinity, and porosity. Bacterial cellulose is pure cellulose, while plant cellulose contains impurities such as hemicellulose and lignin (Pérez and Mazeau, 2004; Gibson, 2012; Lee et al., 2014; Feng et al., 2015). Moreover, plant based cellulose contains a higher fraction of cellulose I_β_ and is less crystalline (Atalla and VanderHart, 1984; Lee et al., 2014; Feng et al., 2015). In general, the microfibrils of bacterial cellulose are smaller than those of plants; consequently, the bacterial cellulose is highly porous and exhibits extensive water retention (Lu and Jiang, 2014; Feng et al., 2015). It should be noted that these are general statements and the actual physical parameters of each material are influenced by many factors, not just the choice of source material. Notably, the growth medium and production method (static vs. agitated vs. bio reactor/trickling bead method) lead to different nanoscale arrangements of microfibrils (Pérez and Mazeau, 2004; Jozala et al., 2014; Lu and Jiang, 2014). Different strains and culture conditions produce different structures, mechanics, morphologies, crystallinity, and pore sizes (Bi et al., 2014; Lu and Jiang, 2014; Luo et al., 2014; Feng et al., 2015). Cellulose is a diverse material as evidenced by the wide range of physical properties. The microfibril formation and crystallization can be adjusted by changing the culture conditions and the source organism (Bi et al., 2014).

### Mechanical Properties

**Figure 4:**
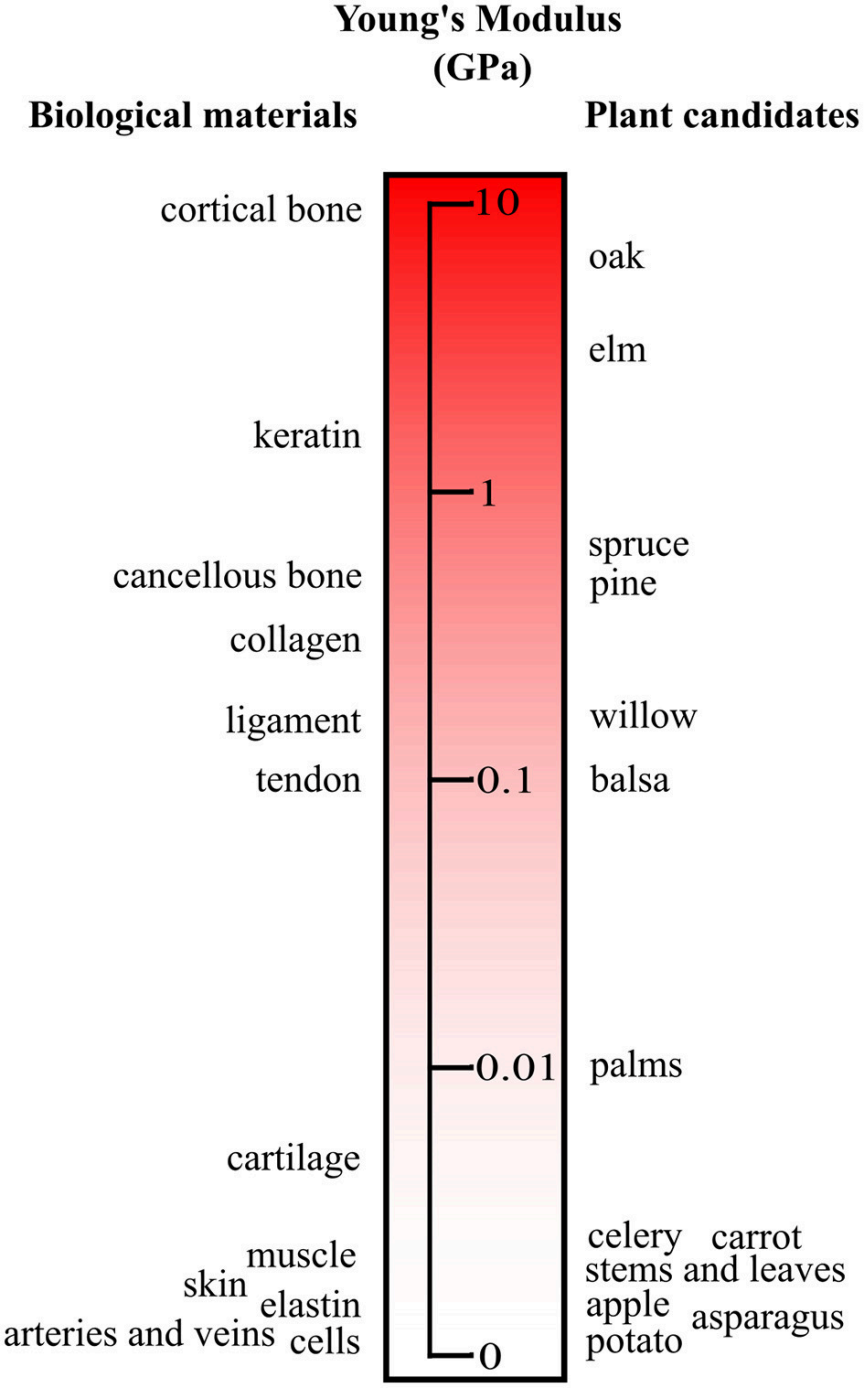
Young's modulus of plant materials and human tissues. A small subset of plant candidates are compared to key biological tissue stiffnesses. The source material can be selected to match the elasticity of the native tissue. It should be noted that with processing and modifications the moduli of the plant candidates can be tuned. Adapted from Gibson et al. (2010) with permission from Cambridge University Press.

The relatively high Young's modulus of bacterial cellulose is attributed to the super-molecular nanostructure (Yamanaka et al., 1989; Feng et al., 2015). The thinner ribbon structures compared to plant based and synthetic fibers are formed through intra- and inter-hydrogen bonding (Yamanaka et al., 1989; Feng et al., 2015). For instance, bacterial cellulose sheets can have a Young's modulus greater than 15GPa as well as a tensile strength of 250MPa (Yamanaka et al., 1989). The extensive hydrogen bonding leads to the high thermal stability, tensile strength, and Young's modulus (Feng et al., 2015). Moreover, these materials have good consistency and viscosity, as evidenced by rheological analysis (Feng et al., 2015). Comparatively, plant-based cellulose also has a vast range of mechanical properties and porosities (Gibson, 2012). Although modifications are feasible (Modulevsky et al., 2014, 2016; Fontana et al., 2017; Gershlak et al., 2017; Hickey et al., 2018), the mechanical and physical properties can be selected by choosing specific source materials (Gibson, 2012) (Figure 4). As cellulose is abundant in nature, the enumerations of different mechanical and physical parameters are extensive (Figure 4). Recently, it was shown that the existing structures of plant tissue can be exploited and repurposed for tissue engineering (Modulevsky et al., 2014, 2016; Fontana et al., 2017; Gershlak et al., 2017; Hickey et al., 2018). This new angle on biomaterial design allows for the intricate structures of plant tissue that have been optimized for analogous functions through years of evolution to be selected for applications of interest. The nanoscale features of cellulose-based materials, both naturally derived and synthetic, can be chosen for specific biological and mechanical functions (Lee et al., 2014). These nanoscale features are integral components of the macroscopic 3D biomaterial as they dictate cellular form and function.

## Scaling up to 3D Macrostructures With Specific Nano- and Micro-Features

### Engineering Materials With Features on Different Length Scales

The challenge of engineering biomaterials with high efficacy is incorporating particular features on the nano-, micro-, and macro-scale. Cellulose materials are highly attractive because of the customizability and control over the features at all levels (Stumpf et al., 2018). At the nanoscale, different crystallinities can be obtained; moreover, the chemical structure of the cellulose can be modified to include specific functional groups to elicit particular cellular responses (He et al., 2014; Shao et al., 2017; Courtenay et al., 2018a,b; Stumpf et al., 2018). For example, collagen can be chemically attached to the cellulose scaffold via linker molecules such as succinic acid (Ribeiro-Viana et al., 2016). At the microscale, the porosities of the materials can be tuned to suit the intended application. In addition, hydrogels and other composites can be created to increase the functionality (Courtenay et al., 2018b; Hickey et al., 2018; Stumpf et al., 2018). On the macroscale, specific structural components and arrangements are required for proper tissue function. Bacterial and synthetic cellulose are often molded or fabricated into the desired configurations (Entcheva et al., 2004; Li et al., 2015; Zang et al., 2015). Importantly, guided assembly-based biolithography (GAB) is a molding technique used to transfer nanoscale functional topographies to the surface of the cellulose (Bottan et al., 2015). The mold is introduced at the gas/liquid interface where the cellulose is being synthesized, and the cellulose nanofibers are directionally assembled in a three-dimensional network dictated by the mold (Bottan et al., 2015). Significantly, the 3D macrostructure of bacterial and synthetic cellulose can be controlled (Bi et al., 2014; Jozala et al., 2014; Bottan et al., 2015; Laromaine et al., 2018). For example, free standing, biocompatible hollow spheres and lenses with porous BC membranes can be synthesized (Laromaine et al., 2018); the control over the geometry is attained through tuning and patterning the hydrophobicity of the synthesis surface (Laromaine et al., 2018). Conversely, for plant derived scaffolds, the complex pre-existing 3D structures can be selected from nature and subsequently modified to suit the application of the biomaterial (Gibson, 2012; Lee et al., 2014; Modulevsky et al., 2014, 2016; Fontana et al., 2017; Hickey et al., 2018). The plant derived scaffolds, as in the case of bacterial and synthetic cellulose, can be tuned chemically and physically, are biocompatible, exhibit vascularization, and are widely available and feasibly produced (Gibson, 2012; Lee et al., 2014; Modulevsky et al., 2014, 2016; Fontana et al., 2017; Hickey et al., 2018).

**Figure 5:**
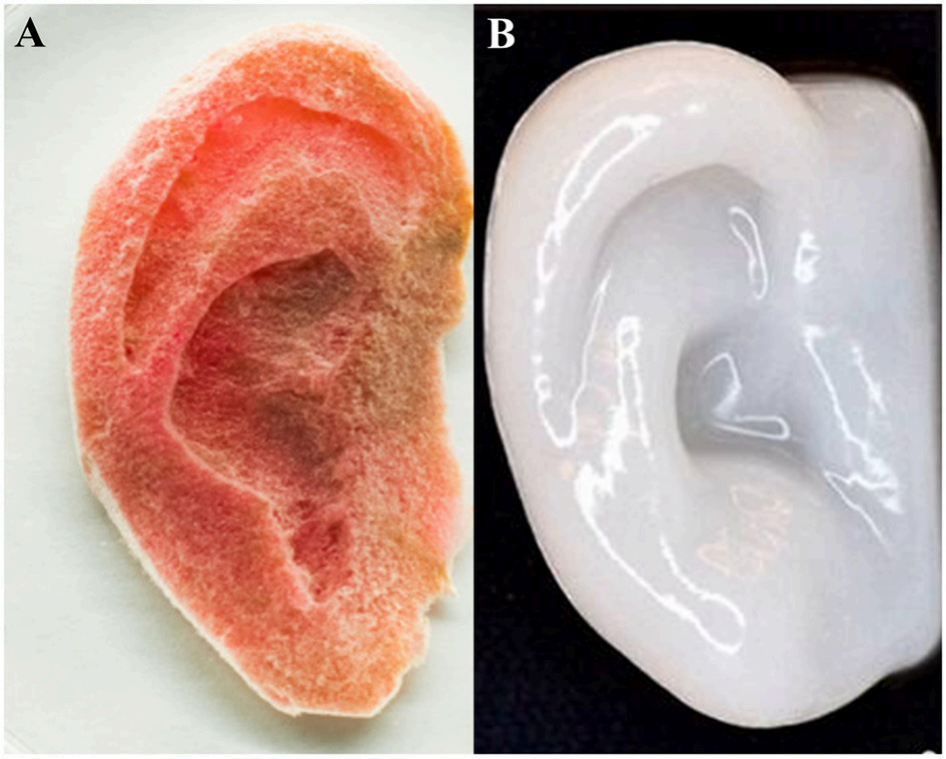
Human ear scaffolds carved out of plant based cellulose **(A)** and 3D printed with nanofibrillated cellulose both cultured with human cells (HeLa and chondrocytes, respectively). **(A)** (Hickey et al., 2018), Copyright 2018. Reproduced with permission from American Chemical Society. **(B)** (Markstedt et al., 2015), Copyright 2015. Reproduced with permission from American Chemical Society.

As a result, there are vast production methods available for producing 3D cellulose scaffolds engineered to have specific features at the nano-, micro-, and macro-scale. The advantage to the molding and fabrication approach is having control over the design particular structures; the advantage to exploiting the existing structures in nature is the high complexity. Combining both approaches opens up even more possibilities and potential applications (Modulevsky et al., 2014, 2016; Fontana et al., 2017; Gershlak et al., 2017; Hickey et al., 2018). Figure 5 depicts two approaches to create macroscopic ear structures: carving and 3D printing/molding.

### Characterization of Nano- and Micro-Features in Macrostructures

Interestingly, incorporation of deuterium has been shown to have no significant differences in the molecular and morphological properties of bacterial cellulose (Bali et al., 2013). Consequently, small angle neutron scattering (SANS) methods can be used to probe cellulose structure and dynamics in addition to conventional techniques (Bali et al., 2013). Furthermore, Lee et al. have shown that the non-centrosymmetry and phase synchronization requirements of vibrational sum frequency generation (SFG) spectroscopy can be used to decipher the 3D organization cellulose of plants, tunicates, and bacteria (Lee et al., 2014). In plant cell walls, this signal is unique to cellulose, as all other matrix polymers in plant cell walls such as hemicellulose, pectin, and lignin are amorphous and do not produce detectable SFG signals (Lee et al., 2014). The cellulose structure and packing have been investigated on the mesoscale of plant cell walls, tunicate tests, and bacterial films (Lee et al., 2014). Armed with the knowledge of the characteristics of the cellulose material at each length scale of interest, researchers can design complex biomaterials for specific applications. In the subsequent sections, we highlight several key applications of these constructs.

## Applications

### Skin and Wound Dressings

Significant interest in using cellulose biomaterials for artificial skin and wound dressings stems from the tunable mechanical properties, high biocompatibility, versatile and customizable surface structure and chemistry, drug releasing capabilities, and moisture maintenance. As a result, several artificial skin products are commercially available.

As such, topical features are required to guide cell infiltration, proliferation, and angiogenesis (Bottan et al., 2015). These topical features of cellulose materials can be conferred with GAB methods (Bottan et al., 2015). In another approach, nanocellulose can be used as a bioink for printing and modifying film surfaces (Rees et al., 2015). In this regard, chemically modified nanocellulose fibrils reduce the viscosity and yield a bioink with suitable rheological properties for printing and skin applications (Rees et al., 2015). The bioprinting allows for the construction of porous nanocellulose structures (Rees et al., 2015). For instance, C-Periodate nanocellulose has been used to print highly porous, 3D track structures with the capacity to carry and release antimicrobial components (Rees et al., 2015).

In an attempt to recreate the complexity of the *in vivo* nanoenvironment, electrospining has also been employed (Liu et al., 2012; Vatankhah et al., 2014). This technique allows for the creation of 3D porous matrices that mimic the natural structure of skin (Liu et al., 2012; Vatankhah et al., 2014). Of particular interest is the electrospinning of composites of cellulose acetate and hydrogels such as gelatin and poly urethane to form the scaffold (Liu et al., 2012; Vatankhah et al., 2014). The addition of the hydrogel can change nanoscale features of the cellulose material such as fiber diameter (Vatankhah et al., 2014). Moreover, the porosity, stiffness, hydrophilicity, fluid uptake, and surface area can be tuned by varying the ratio of the constituents to increase the rate of wound healing (Liu et al., 2012; Vatankhah et al., 2014). One common issue in designing artificial skins and wound dressings is that the material must adhere to the wound to support healing but then must be easily removed without damaging the regenerated tissue (Vatankhah et al., 2014). Varying the relative amounts of constituents in composites can achieve the desired adherency features of the material (Vatankhah et al., 2014). Notably, electrospun cellulose acetate/gelatin composites at a ratio of 25:75 promote cell proliferation and collagen deposition, while a ratio of 75:25 can act as a low-adherent wound dressing (Vatankhah et al., 2014).

Nanofibrillar cellulose has also been implicated in clinical trials (Hakkarainen et al., 2016). Functionalized nanofibrillar cellulose dressings have been used to heal and regenerate skin for burn victims (Hakkarainen et al., 2016). The physical and mechanical properties of the nanocellulose dressings can be optimized to suit the patient's needs (Hakkarainen et al., 2016). Hakkarainen et al. demonstrated that functionalized cellulose dressings can be superior to the existing commercially available products such as Suprathel® (Hakkarainen et al., 2016). Epithelialized skin regeneration and a lack of inflammatory response to the cellulose dressing were observed (Hakkarainen et al., 2016). The dressing attaches easily to the wound, yet detaches on its own after skin regeneration is completed (Hakkarainen et al., 2016). Although the dressing itself was not antibacterial, it did not promote bacterial growth (Hakkarainen et al., 2016). Bacterial nanocellulose is biocompatible and has been applied to full-thickness skin defect models (Fu et al., 2013). Using these porous membranes stimulates an increase in the healing rate along with a decrease in inflammation (Fu et al., 2013).

During the synthesis of the cellulose materials, the pore size can vary with the thickness of the membrane (Fu et al., 2013; Li et al., 2015). For example, the bottom side of BC films has a looser and rougher structure than the top side (Li et al., 2015). It has been shown that the increased porosity improved the wound healing rate and reduced the inflammatory response compared to control gauze and the more dense top side, as cell migration and diffusion were more permissible (Li et al., 2015). The less porous top side was more effective in preventing infection and water-loss (Li et al., 2015). Polyvinyl alcohol (PVA)/cellulose nanowhisker nanocomposite hydrogels have also been applied to wound healing applications (Gonzalez et al., 2014). Including nanowhiskers endows greater control over the physical properties of the hydrogels (Gonzalez et al., 2014). Specifically, the porosity can be tuned; the presence of the cellulose nanowhiskers decreases the pore size, but it does not affect the gel formation process (Gonzalez et al., 2014). Adding cellulose nanowhiskers mechanically reinforces the composite materials (Gonzalez et al., 2014). In the context of the skin application, the presence of nanowhiskers does not increase the drying rate beyond the *in vivo* optimal range (Gonzalez et al., 2014). The composite materials offer protection from bacterial invasion as well (Gonzalez et al., 2014).

As bacterial cellulose alone does not exhibit antibacterial properties, and infection prevention is vital for wound healing applications, antimicrobial agents such as octenidine and minocycline have been combined with cellulose biomaterials (Moritz et al., 2014; Bajpai et al., 2015). For the use of thin films, a Fickian diffusion model is applicable; however, the swelling of the polymer often results in non-Fickian drug diffusion dynamics (Moritz et al., 2014; Wu et al., 2014a,b; Bajpai et al., 2015). The scaffold thickness, surface area to volume ratio, structure, and chemistry at the nanoscale influence the diffusion and release of the drugs (Liu et al., 2012; Moritz et al., 2014; Bajpai et al., 2015). Mortiz et al. demonstrated that incorporating octenidine did not alter the mechanical properties or stability; nevertheless, this assumption cannot be assumed for different drugs or production methods (Moritz et al., 2014). Combining cellulose nanowhiskers with hydrogels is an effective method of tuning the physical characteristics and drug release properties (Bajpai et al., 2015). By adding the nanoscale cellulose crystals, higher control over the drug release is obtained (Bajpai et al., 2015). These composite materials did not exhibit thrombogenesis or hemolysis (Bajpai et al., 2015). Conversely, protein adsorption, antibacterial, and antifungal properties were observed (Bajpai et al., 2015). When combined with antimicrobial agents, the resultant material is viable to cells, is antibiotic, and induces a low inflammatory response (Liu et al., 2012; Moritz et al., 2014; Bajpai et al., 2015). Another approach to prevent bacterial infection is to incorporate silver nanoparticles (Wu et al., 2014a,b). In particular, silver nanoparticles were generated and self-assembled on the surface of cellulose nanofibers (Wu et al., 2014a,b). These materials are antibacterial and enable the proliferation of cells with low cytotoxicity (Wu et al., 2014a,b). In addition, these materials have been applied to wound models (Wu et al., 2014a,b). Significantly, these dressings regenerated epidermal and dermis more effectively than untreated wounds (Wu et al., 2014a,b).

### Bone Tissue

As a consequence of the versatility of cellulose, these biomaterials can be adapted to be applicable to the stiff and mechanically demanding environment of bone (Kim et al., 2014; Stumpf et al., 2018; Torgbo and Sukyai, 2018).

As discussed in the skin and wound healing section, templating the biomaterial structure is a viable approach used to build biomimetic constructs. In the context of bone, it has been shown that a reverse templating method can be used to create gyroidal cellulose scaffolds (Torres-Rendon et al., 2015). This approach allows researchers to mathematically define and control pore geometries (Torres-Rendon et al., 2015). As this review stresses, the nanoscale details dictate macroscopic properties; therefore, bottom-up methods of creating 3D scaffolds are instrumental.

In contrast to templating, a popular method of creating nanocomposites for bone tissue replacements is electrospinning. As the mechanical properties of hydrogels are insufficient for withstanding the physical stress exerted on bones, they are often fortified with nanocellulose (Zhou et al., 2013; Eftekhari et al., 2014; Rescignano et al., 2014; Zhang et al., 2015). For example, cellulose nanocrystals can act as physical supports to electrospun matrices of poly lactic acid (PLA) and poly vinyl alcohol (PVA) hydrogels (Zhou et al., 2013; Eftekhari et al., 2014; Zhang et al., 2015). Modifying the surface chemistry with strategies such as maleic anhydride grafting, PEG grafting, and sodium dodecyl sulfate (SDS) improves the interfacial adhesion between the cellulose and PLA along with the tensile strength (Eftekhari et al., 2014; Zhang et al., 2015). Moreover, the nanocrystals reduce the diameter and polydispersity of the matrix fibers (Zhou et al., 2013). The mechanical and thermal stability increases with the addition of the cellulose nanocrystals (Zhou et al., 2013). These scaffolds have a tensile strength >10 MPa and are biocompatible (Zhou et al., 2013; Zhang et al., 2015). The electrospun nanofibers with different weight ratios can be used to produce biomimetic bone structures (Chalal et al., 2014).

Natural bone is highly porous; therefore, methods of creating highly porous biomimetic materials for bone tissue engineering are integral (Rodríguez et al., 2014). One approach of introducing pores is laser ablation of cellulose acetate electrospun fibers (Rodríguez et al., 2014). Pore sizes ranging from 50 to 300 μm can be fabricated without affecting the surround material (Rodríguez et al., 2014). These constructs can be further processed on the nanoscale to become mineralized to an extent that resembles *in vivo* hydroxyapatite levels (Chalal et al., 2014; Rodríguez et al., 2014). The porous mineralized scaffolds increase osteoblast attachment and cell density at the pore sites (Rodríguez et al., 2014).

Natural bone consists mainly of collagen and minerals similar in composition to hydroxyapatite (Li et al., 2012). Mimicking this complex composition is essential for bone tissue engineering. Cellulose nanofibers/hydroxyapatite composites can be used to emulate natural bone, namely the compressive strength (0.1–12 MPa), compressive modulus (6–330 MPa), porosity, and biocompatibility (Li et al., 2012; Eftekhari et al., 2014; Garai and Sinha, 2014; Park M. et al., 2015; Huang et al., 2017). The proper dispersion of hydroxyapatite is required to emulate the natural environment (Park M. et al., 2015). In the absence of other composites, hydroxyapatite aggregates and precipitates; hence, the colloidal stability must be increased prior to its use in 3D scaffolding materials. Cellulose oxidation with compounds such as 2,2,6,6-tetramethylpiperidine-1-oxyl (TEMPO) can be used to accomplish the desired dispersion (Park M. et al., 2015). The oxidation yields negatively charged nanofibres onto which the hydroxyapatite adsorbs and creates a hydrogel that can be crosslinked (Park M. et al., 2015). The modified cellulose structure yields a highly porous bioactive material (Li et al., 2012; Huang et al., 2017). The mineralization of the macroporous scaffolds results in an environment resembling native bone tissues' mineralized ECM both topographically and chemically (Sundberg et al., 2015). Messenchymal stem cells can proliferate and differentiate toward osteoblasts on these scaffolds, confirming the material as a potential candidate for use in bone tissue engineering (Park M. et al., 2015; Sundberg et al., 2015; Huang et al., 2017). *In vivo* studies involving cellulose scaffolds combined with gelatin hydrogels that were subsequently coated with hydroxyapatite revealed that this approach enhanced new bone formation (Huang et al., 2017).

Bone implant integration is a major concern in the field of bone tissue engineering. In an attempt to improve integration of implants, cellulose alternatives to conventional ceramic and metal implants have been proposed. The surface functionalization with 45S5 bioactive glass individually wrapped and interconnected with fibrous cellulose nanocrystals was deposited on 316L stainless steel (Chen et al., 2015b). Rapid mineralization including hydroxapatite occurred in the presence of simulated body fluid (Chen et al., 2015b). The mineralized scaffold expedited cell attachment, spreading, proliferation, differentiation, and ECM mineralization, showing cellulose-based implants are a promising alternative to conventional methods that are not viable long term (Chen et al., 2015b).

Carbon nanotubes (CNTs) have many potential applications in biology; however, a significant challenge is introducing them into a suitable 3D structure (Park S. et al., 2015; Gutiérrez-Hernández et al., 2017). Furthermore, similar to the issue with the hydrophobic hydrogels and the hydroxyapatite, the CNTs tend to aggregate together. To circumvent this effect, an amphiphilic comb-like polymer (APCLP) can be adsorbed onto CNTs. *In situ* hybridization of CNTs coated with an APCLP with cellulose produces a homogeneous 3D microporous structure that is osteoconductive and osteoinductive (Park S. et al., 2015; Gutiérrez-Hernández et al., 2017).

As cellulose fibers resemble the collagen fibers of bone tissue, cellulose has been implicated in bone tissue engineering applications (Shi et al., 2012). In particular, bacterial cellulose can serve as a localized delivery system to increase the local concentration of cytokines (Shi et al., 2012). It has been shown that the biocompatible scaffolds supported osteodifferentiation in the presence of bone morphogentic protein 2 (BMP-2) (Shi et al., 2012). Greater *in vivo* bone formation and calcium deposition was stimulated with BMP-2 loading (Shi et al., 2012). Likewise, cellulose nanocrystal—hydrogel composites can be implicated in the transport bioplymeric nanoparticles to bone marrow (Rescignano et al., 2014).

Although further investigation is required to uncover the full potential of cellulose-based materials for bone tissue engineering, the current body of work contests that cellulose materials present a promising approach to solving a major biomedical issue. Significantly, cellulose membranes have been shown to guide bone regeneration *in vivo* (Lee et al., 2017).

### Neural Applications

Cellulose scaffolds are a suitable material for 3D nerve cell proliferation and differentiation because of the adjustable surface chemistry and mechanical/physical properties (Innala et al., 2014; Jonsson et al., 2015). Chemical modification and protein coating of cellulose materials can be used to enhance integrin based attachment and cell—scaffold interactions (Innala et al., 2014; Jonsson et al., 2015).

Nerve tissue engineering presents an issue that is unique to a subset of cell types including neurons and myocytes: electrical stimulation. As a result, electoactive, flexible, 3D nanostructured biomaterials are required. To satisfy these criteria, cellulose scaffolds coated with conductive materials such as poly (3,4-ethylenedioxythiophene) (PEDOT) and multi-walled carbon nanotubes, or carbonization can be used (Chen et al., 2015a; Kuzmenko et al., 2016). Such materials have tunable pore sizes, mechanical properties, and electrical conductivities; moreover, they are biocompatible and foster neural differentiation (Chen et al., 2015a; Kuzmenko et al., 2016).

It is often desirable to incorporate growth factors into the surrounding nano- and micro-environments of stem cells (Wang et al., 2013; Kandalam et al., 2017). Recently, cellulose bases scaffolds have been used to transport and release growth factors to guide neural differentiation and repair damaged tissue caused by strokes (Wang et al., 2013; Kandalam et al., 2017). Pharmacologically active microcarriers (PAMs) and stem cells can be delivered via cellulose-based biomaterials including scaffolds and injectable gels (Wang et al., 2013; Kandalam et al., 2017). Different release profiles, namely biphasic dynamics, of drugs can be designed by tuning the properties of the cellulose construct (Wang et al., 2013; Kandalam et al., 2017). Similarly, growth factor delivery in the context of spinal cord injuries has been studied using tubular cellulose composite materials (Hackett et al., 2010). Cellulose biomaterials implicated in spinal cord injury have been shown to promote the regeneration of neurons (Tsai et al., 2006).

In addition to growth factor loading, drug loading has important implications in psychiatry (Naseri-Nosar et al., 2017). Loading cellulose-based biomaterials with drugs is a promising avenue for drug delivery (Naseri-Nosar et al., 2017). The tunable mechanical properties, highly porous structure, adjustable stability, and excellent biocompatibility make cellulose an ideal candidate for nerve tissue repair and drug delivery systems (Wang et al., 2013; Du et al., 2014; Min et al., 2015; Kuzmenko et al., 2016; Naseri-Nosar et al., 2017). Significantly, it has been shown that cellulose constructs can be used as nerve guidance conduits for sciatic nerve defects in rats (Naseri-Nosar et al., 2017). The results of this study revealed that citalopram-loaded cellulose materials can mediate the functional recovery of the sciatic nerve (Naseri-Nosar et al., 2017).

### Blood Vessels

The two most commonly used vascular graft materials are expanded polytetrafluoroethylene (ePTFE) and poly(ethylene terephthalate) (PET). Despite the high success rate of these materials, their applicability to small vessels is limited due to thrombosis (Esguerra et al., 2010; Fink et al., 2011). As such, there is a need for blood compatible materials with appropriate biochemical and physical properties for vasculature engineering (Esguerra et al., 2010; Fink et al., 2011). In comparison to conventionally-used graft materials, bacterial cellulose constructs exhibit no significant difference in platelet consumption and coagulation, as compared with PET, ePTFE, and heparin coated PVC (Fink et al., 2011). However, it should be noted that the complement activation parameters sC3a and sC5b-9 were much higher for BC, as compared with the other materials for both 4 and 6 mm tubes diameter tubes (Fink et al., 2011). In addition, an *in vivo* model using hamsters demonstrates the high biocompatibility and low immune response to these materials (Esguerra et al., 2010). Likewise, *in vivo* implantation of a bacterial cellulose blood vessel in the carotid arteries of sheep showed epithelial cell coverage and patency for up to 13 months (Malm et al., 2012). Nevertheless, the patency of the unmodified structures used in this study was inconsistent (Malm et al., 2012). On the contrary, bacterial cellulose blood vessels molded in oxygen permeable polydimethylsiloxane (PDMS) templates yield appropriate mechanical properties and high stability (Zang et al., 2015). These vessels have been successfully implanted into rabbit femoral arteries, and endothelialization was observed (Zang et al., 2015).

In order to improve the adhesion of human microvascular endothelial cells (HMEC) to cellulose grafts, chimeric proteins containing both a cellulose-binding domain and an adhesion peptide motif can be incorporated (Andrade et al., 2010). The recombinant proteins improve both the attachment and spreading of HMECs on the cellulose grafts (Andrade et al., 2010). Blood vessels are complex structures that not only act as a transport system, but also involve the transvascular migration of different cell types and molecules (Wang et al., 2015). Simulated vascular lumens consisting of human umbilical vein endothelial cells (HUVECs) and a cellulose/collagen scaffold can replicate the transvascular migration and hemodynamics of native vessels (Wang et al., 2015).

Nanocomposite materials consisting of nanocrystalline cellulose and fibrin are applicable to small-diameter replacement vascular grafts (SDRVGs) (Brown et al., 2013). Chemical attachment of fibrin to the cellulose can be mediated and tailored with periodate oxidation of the cellulose (Brown et al., 2013). The nanocrystalline cellulose provides the elastic hydrogel with rigidity. Interestingly, the maximum strength and elongation of the composites were comparable to those of native blood vessels (Brown et al., 2013). Similarly, a composite material of cellulose nanowhiskers and cellulose acetate propionate can be used as an alternative to conventional synthetic blood vessels (Pooyan et al., 2012). The nanowhiskers act as reinforcements, while the cellulose acetate propionate provides the hydrogel matrix (Pooyan et al., 2012). Resultantly, the percolated structure with improved mechanical properties can withstand the physiological pressure surface features of human blood vessels (Pooyan et al., 2012).

### Other Applications

**Figure 6:**
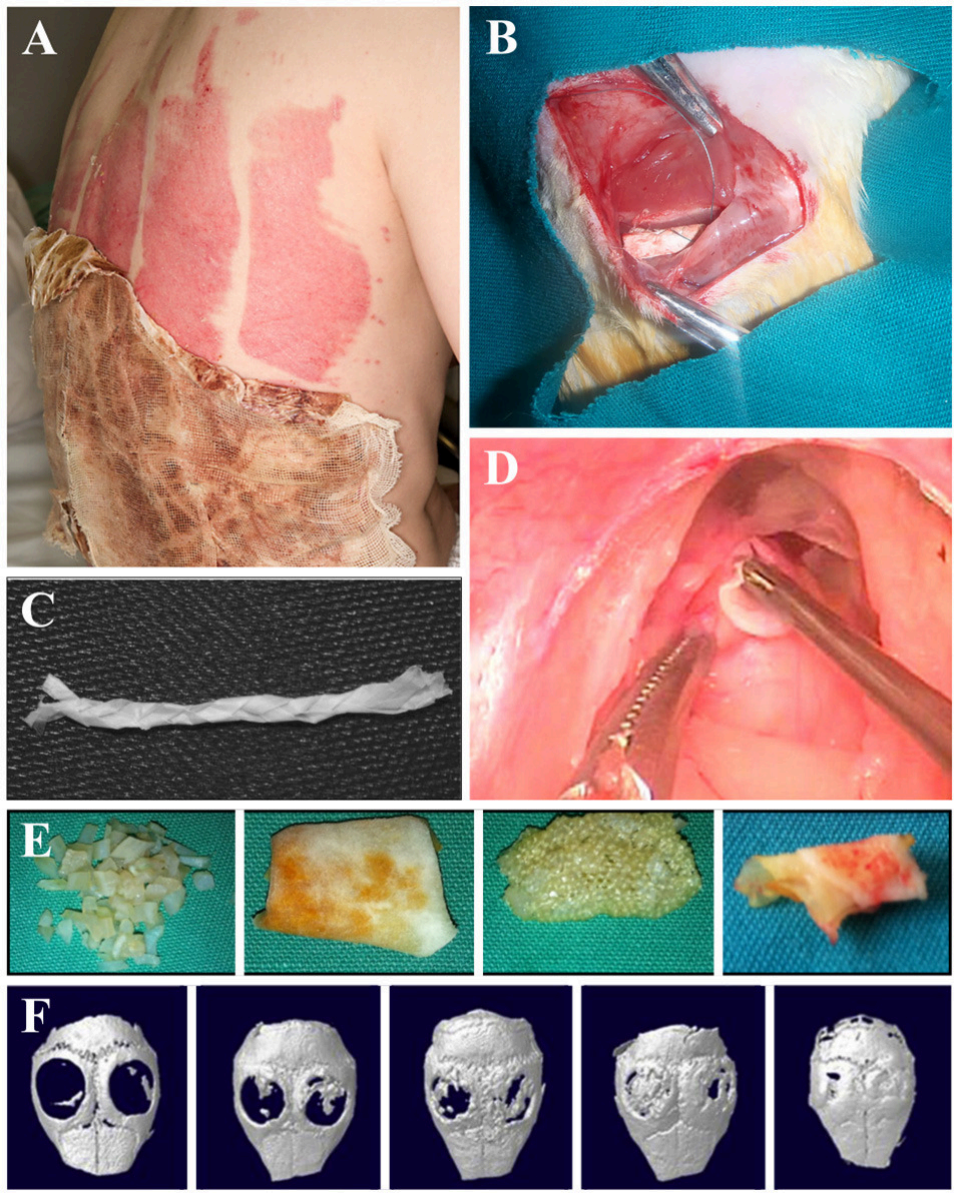
Applications of cellulose biomaterials. **(A)** Skin, **(B)** nerve, **(C)** tendon/ligament, **(D)** larynx, **(E)** cartilage, **(F)** bone. **(A)** (Hakkarainen et al., 2016), Copyright 2016. Reproduced with permission from Elsevier Inc. **(B)** (Naseri-Nosar et al., 2017), Copyright 2017. Reproduced with permission from Elsevier Inc. **(C)** (Mathew et al., 2013), Copyright 2013. Reproduced with permission from John Wiley and Sons. **(D)** (De Souza et al., 2011), Copyright 2011. Reproduced with permission from Elsevier Inc. **(E)** (Guler et al., 2015), Copyright 2015. Reproduced with permission from John Wiley and Sons. **(F)** (Park S. et al., 2015) Copyright 2015. Reproduced with permission from Elsevier Inc.

This review highlights several key applications of cellulose-based materials that have been extensively investigated (Figure 6). The fact that cellulose-based materials can be applied to such a wide range of tissue is a testament to its versatility and adaptability (Figure 6). We stress that the potential uses of cellulose-based materials are not restricted to the categories reviewed here. For example, studies have shown that muscle, tendons/ligaments, cartilage, vertebrae disks, urinary tracts, and larynx tissues are applicable because of the tunable physical and chemical properties of cellulose (Entcheva et al., 2004; Bodin et al., 2010; Hendriks et al., 2010; Borges et al., 2011; De Souza et al., 2011; Dugan et al., 2013; Mathew et al., 2013; Yang et al., 2013; Martínez Ávila et al., 2014; Guler et al., 2015; Markstedt et al., 2015; Yin et al., 2015; Silveira et al., 2016) (Figure 6).

### Remaining Challenges and Future Directions

Although substantial progress has been made in the field of tissue engineering, there are no materials that fully capture the intricacies of the native tissue nor restore function to an ideal level. As a result, the remaining challenges will be to innovate new composite materials with nanoscale engineering methods to produce fully biomimetic tissues. As the complexity of the application increases, such as in highly dynamic tissues, an active remodeling of the scaffolding will be required. Thus, the complex interplay between the cells and the artificial matrix will be paramount.

## Conclusion

In order to recreate fully functional tissue, the biochemical and biophysical properties at the must be designed from the nanoscale up. The nanoscale features dictate cell function and scaffold applicability. Here we have condensed a wealth of knowledge in the field of cellulose-based biomaterials in the context of bottom-up approaches for tissue engineering. Evidently, cellulose-based materials have great potential to become the next generation of standard biomaterials because of their diversity and versatility of biochemical and biophysical characteristics.

## Author Contributions

All authors listed have made a substantial, direct and intellectual contribution to the work, and approved it for publication.

### Conflict of Interest Statement

The authors declare that the research was conducted in the absence of any commercial or financial relationships that could be construed as a potential conflict of interest.
